# Examining the effects of school–vacation transitions on depression and anxiety in adolescents: network analysis

**DOI:** 10.1192/bjo.2024.806

**Published:** 2025-01-17

**Authors:** Yumeng Ju, Yumeng Yang, Rui Yuan, Yafei Chen, Junwu Liu, Wenwen Ou, Yunjing Li, Siqi Yang, Yimei Lu, Liang Li, Mei Huang, Mohan Ma, Guanyi Lv, Xiaotian Zhao, Yaqi Qing, Jin Liu, Yan Zhang

**Affiliations:** Department of Psychiatry, and National Clinical Research Center for Mental Disorders, The Second Xiangya Hospital of Central South University, Changsha, China; Xiangya School of Medicine, Central South University, Changsha, China

**Keywords:** Network analysis, child and adolescent psychiatry, depression, anxiety, symptom network

## Abstract

**Background:**

The school–vacation cycle may have impacts on the psychological states of adolescents. However, little evidence illustrates how transition from school to vacation impacts students’ psychological states (e.g. depression and anxiety).

**Aims:**

To explore the changing patterns of depression and anxiety symptoms among adolescent students within a school–vacation transition and to provide insights for prevention or intervention targets.

**Method:**

Social demographic data and depression and anxiety symptoms were measured from 1380 adolescent students during the school year (age: 13.8 ± 0.88) and 1100 students during the summer vacation (age: 14.2 ± 0.93) in China. Multilevel mixed-effect models were used to examine the changes in depression and anxiety levels and the associated influencing factors. Network analysis was used to explore the symptom network structures of depression and anxiety during school and vacation.

**Results:**

Depression and anxiety symptoms significantly decreased during the vacation compared to the school period. Being female, higher age and with lower mother's educational level were identified as longitudinal risk factors. Interaction effects were found between group (school versus vacation) and the father's educational level as well as grade. Network analyses demonstrated that the anxiety symptoms, including ‘Nervous’, ‘Control worry’ and ‘Relax’ were the most central symptoms at both times. Psychomotor disturbance, including ‘Restless’, ‘Nervous’ and ‘Motor’, bridged depression and anxiety symptoms. The central and bridge symptoms showed variation across the school vacation.

**Conclusions:**

The school–vacation transition had an impact on students’ depression and anxiety symptoms. Prevention and intervention strategies for adolescents’ depression and anxiety during school and vacation periods should be differentially developed.

Depression and anxiety are highly prevalent among adolescents and often cause adverse impacts on their life and academic performance.^1^ A national-scale psychiatric epidemiological survey showed that the prevalence of depression and anxiety disorder among Chinese adolescents was 4.4 and 5.4%, respectively.^2^ A recent meta-analysis also revealed that the global prevalence of clinically significant depression and anxiety symptoms among children and adolescents has increased by approximately 25.2 and 20.5%, respectively.^3^

Previous evidence indicated that the transition from school to vacation can have a significant impact on the mental health of adolescent students. A study has identified marked fluctuations in adolescent depression, anxiety and everyday hassles across school–vacation cycles.^4^ Specifically, depression and anxiety levels increased by at least 50% during school terms compared to vacation periods.^4^ In addition, research found that school problems have adverse effects on treatment responses among adolescents with depression.^5^ Those with school difficulties were five times less likely to respond to antidepressants than those without.^6^ Therefore, school–vacation transition may contribute to the development and maintenance of adolescent depression and anxiety.

Recent studies suggested that mental health problems may be better understood through the interactions of various observable symptoms rather than through the assumption of a single underlying cause.^7^ For example, unlike physical illnesses such as cancer, which can often present a variety of symptoms that can be traced back to a common underlying cause (i.e. tumour), mental health issues like depression may not have a singular or identifiable cause.^7^ Instead, depression may arise from the complex interplay of various symptoms that can reciprocally influence each other and contribute to its onset and progression.^8^ As such, network analysis has emerged as a valuable method for understanding the relationships among observable symptoms or behaviours, which can help us gain a better understanding of mental health issues.^7^ According to the network theory, individual symptoms in the network are represented by nodes, and their interactions are represented by edges between nodes.^7^ Central symptoms refer to the nodes that have strong connections with many other nodes in the network.^9^ Therefore, activation of the central symptoms is more likely to activate other symptoms in the network, leading to the onset and progress of the syndrome.^10^ Targeting the central symptoms may be an effective strategy for preventing or treating the prolonged activation of the overall symptom network, resulting in the alleviation of associated symptoms, and ultimately leading to the relief of mental distress.^11^ Furthermore, there is a high prevalence of co-occurring depression and anxiety in adolescents.^12^ According to symptom network theory, specific nodes can act as bridge symptoms that link two different symptom clusters.^13^ Therefore, depression and anxiety can be activated by one another if they share one or more common nodes (i.e. bridge symptoms) in the network.^13^ Addressing these bridge symptoms may be an important strategy for treating or preventing comorbidity between depression and anxiety in adolescents.

Exploring the complicated and dynamic link between depression and anxiety symptoms during the school–vacation cycle could yield meaningful information for the prevention and intervention of these two types of most common mental distress in adolescents. However, to date, little is known about the interconnected structure of depression and anxiety symptoms over naturalistic school-to-vacation cycles. Only three investigations have conducted network analysis to clarify the symptom structures of depression and anxiety in adolescents.^14–16^ In particular, one study found that ‘Sad Mood’, ‘Irritable’, ‘Worry Too Much’ and ‘Guilty’ were central symptoms in the network, and ‘Guilty’, ‘Sad Mood’ and ‘Suicide Ideation’ were bridge symptoms linking anxiety and depressive symptom clusters.^14^ A longitudinal study identified that ‘Depressed Affect’ was consistently the most central symptom as well as the most important bridge symptom in the network during various periods of the COVID-19 pandemic. This study also revealed that the global strength and the connections between symptoms changed over time.^15^ Another longitudinal study that assessed symptom networks from early childhood to mid-adolescence uncovered developmental changes in the overall structure of the depression–anxiety network. Results revealed that ‘Anxious/Fearful’ and ‘Unhappy/Sad’ were consistently the most central symptoms, and ‘Guilty’, ‘Worries’ and ‘Cries’ were common bridge symptoms throughout the course of development.^16^ Nonetheless, the relationship between depression and anxiety symptoms, as well as the importance of specific symptoms, varied significantly over this developmental period (5–14 years).^16^ Although findings are inconsistent among the abovementioned studies (probably because of the heterogeneity of participants and measurements), these results supported that the symptom network of depression and anxiety would change dynamically over time in adolescents, especially under the influence of external factors.^7,15,16^ As one of the prevalent and major external factors influencing the mental health status of students, the school–vacation cycle may influence the intercorrelation between depressive and anxious symptoms. To date, however, no research has investigated the variation in the interactive pattern of depression and anxiety symptoms among adolescents during the school year compared to during vacation.

Therefore, our study aimed to examine the symptom network structures of depression and anxiety during school and vacation periods in adolescent students. In addition, we sought to investigate the changes in depression and anxiety levels and the related factors during school and vacation periods by using multilevel mixed-effect models (MLMs). We hypothesised that the depression and anxiety symptom severity as well as the depression–anxiety symptom network structure would significantly differ between school and vacation periods. Results of this study aim to provide insights into the effect of school–vacation transitions on depression and anxiety in adolescents during the school and vacation periods, and help inform the development of targeted interventions and mental health services for the adolescents.

## Method

### Participants and procedures

Participants were recruited by convenience sampling through the ‘Evergreen Mental Health Promotion Plan’, a school-based programme to improve the screening and treatment of depression and anxiety in adolescent students.^17^ The survey was conducted in one to three classes within each school, aiming to achieve a relatively representative sample. Participants were from 14 middle schools located in six urban districts in Changsha, Hunan Province, China. Students who were aged 12–18 years old with parental consent to participate in our study who could complete all scales and assessments (as in the Measures section) independently were included in the study. Students were not quarantined during either period and were in regular classes or on vacation in both surveys. The baseline survey was conducted during the school year from 12 February 2021 to 12 May 2021. Data were collected through a self-administered paper-based questionnaire anonymously, and a total of 1501 participants completed the questionnaire survey. A well-trained psychiatrist or psychologist informed the participants about the aims and procedure of the study before the survey. A follow-up study with similar contents and procedures was conducted during the summer vacation from 9 August 2021 to 22 September 2021. The 1501 participants were re-invited to participate in the survey via a website link, and all answers were transferred and stored into the electronic data capture (EDC) system of the Second Xiangya Hospital. Incomplete questionnaire entries or entries with obvious errors were excluded during data analyses.

#### Ethics and consent

The authors assert that all procedures contributing to this work comply with the ethical standards of the relevant national and institutional committees on human experimentation and with the Helsinki Declaration of 1975, as revised in 2008. All procedures involving human participants were approved by the Ethics Review Committee of the Second Xiangya Hospital of Central South University (approval number 004). Written informed consent was obtained from students’ legal guardians at the beginning of the study.

### Measures

#### Demographic characteristics

Social demographic data included gender (male 1, female 2), age (years), residence (urban 1, rural 2), being the only child in a family (yes 1, no 2), parental educational level (primary school or below 1, middle school 2, high school 3, bachelor degree 4, master degree or above 5) and monthly household income per capita (<1000 CNY 1, 1000–5000 CNY 2, 5000–10 000 CNY 3, 10 000–50 000 CNY 4, > 50 000 CNY 5).

#### Depression

Depressive symptoms were assessed using the Chinese version of the Patient Health Questionnaire-9 (PHQ-9).^18^ The scale consisted of nine items asking about the frequency of the nine depressive symptoms during the past two weeks. Items were scored on a Likert scale ranging from ‘0’ (not at all) to ‘3’ (nearly every day). The PHQ-9 has shown good validity and reliability among Chinese adolescents.^19^ A PHQ-9 total score of 0–4 points indicates no depression, 5–9 indicates mild depression, 10–14 indicates moderate depression and 15–27 indicates severe depression. A score of 10 or higher on the PHQ-9 indicates the presence of clinically significant depression, suggesting a probable diagnosis of depression.

#### Anxiety

Anxiety symptoms were examined by the Chinese version of the Generalized Anxiety Disorder 7-item (GAD-7) questionnaire,^20^ which consisted of seven questions measuring the frequency of seven anxiety symptoms in the past two weeks. Each question in the GAD-7 questionnaire was scored on Likert scale ranging from ‘0’ (not at all) to ‘3’ (nearly every day). The GAD-7 questionnaire also demonstrated good validity and reliability in Chinese adolescents.^21^ A GAD-7 Questionnaire total score of 0–4 points indicates no anxiety, 5–9 indicates mild anxiety, 10–14 indicates moderate anxiety and 15–21 indicates severe anxiety. A score of 10 or higher on the GAD-7 questionnaire indicates the presence of clinically significant anxiety, suggesting a probable diagnosis of anxiety.

### Statistical analysis

#### Data distribution and general difference

Data distribution was assessed by a probability plot and Kolmogorov–Smirnov test. Continuous variables with normal distribution were presented as the mean and standard deviation. Nonnormal variables were reported as median and interquartile ranges. Categorical variables were presented as frequency and percentages. The difference in continuous variables with nonnormal distribution between groups was detected by the Mann–Whitney *U*-test. The difference in continuous variables with normal distribution between groups was tested by analysis of variance (ANOVA). Categorical variables were compared between groups using the chi-square test.

#### Multilevel mixed-effect models

The effect of the school–vacation cycle and participants’ characteristics on depression and anxiety symptoms was measured by MLMs using the R package lmerTest (version 3.1-3, R Core Team, Vienna, Austria; https://cran.r-project.org/web/packages/lmerTest/). The total scores on the PHQ-9 or GAD-7 questionnaire were treated as the outcome variable. The group (school versus vacation) variable (Level 1) and participants’ characteristics (Level 2) served as multiple predictors. The group variable was coded as 1 or 2 to represent the school and vacation time points, respectively. To investigate whether depression and anxiety symptom changes were differently influenced by factors across different time points, we repeated the MLM analysis by adding the interaction terms (group * participants’ characteristics). The regression analysis was repeated using each category of the categorical variable as a reference category. The interactive effect between group and the factors was checked for significance. Unstandardised coefficients (*b*) and standard errors for regression models were calculated to describe the association of each independent variable with the outcome variables.

#### Network estimation and stability analysis

We estimated the network structure using the Gaussian graphical model (GGM) via the R packages ‘qgraph’ (https://cran.r-project.org/web/packages/qgraph/index.html) and ‘bootnet’ (https://cran.r-project.org/web/packages/bootnet/index.html). The networks were composed of 16 nodes that were represented by the 16 items from PHQ-9 and GAD-7 questionnaire (Supplemental Table 1 available at https://doi.org/10.1192/bjo.2024.806). A regularised network was fit by the EBIC glasso function, as previous research found that regularised network estimators achieved the best sensitivity and specificity in edge weights at the sample size of *n* = 1000.^22^ Rank transformations (Spearman correlations as input) were applied as the data were skewed.^22^ The Fruchterman–Reingold algorithm was applied to create the network graph.^23^ The thickness of the edge was represented by the regularised partial correlations coefficient (i.e. edge weight) between the two nodes. Before demonstrating network metrics and conducting network comparison analysis, network accuracy and stability were assessed by the bootstrapping procedure. In addition, the edge weights and centrality index differences from one another were tested for significance (see Supplemental Information for more details).

#### Network centrality indices

We computed the centrality indices, including global strength, nodal strength and bridge strength, as a recent study indicated that closeness and betweenness might not be directly interpretable in the psychological network.^24^ Global strength was defined as the sum of the absolute values of all edges in the network. Higher global strength indicates a more strongly connected network, which was suggested to be related to an easier activation of the network and the persistence of the syndromes.^7^ Nodal strength was calculated by summing the absolute values of all the edges of a given node with all other nodes in the network. Higher nodal strength indicates a more central position of the symptom in the network. Such nodes might be priority prevention and treatment targets.^10^ The bridge strength was calculated by summing the absolute values of all the edges of a given node with all the nodes in other symptom clusters.^13^ Higher bridge strength indicates a higher connection of the symptom with other disorders. Such nodes may be preferred targets for the prevention and treatment of psychiatric comorbidity.^13^

#### Network comparison

The difference in network properties (including edge weights and centrality indices) between groups was assessed by the Network Comparison Test (NCT) R package, a two-tailed permutation test. We conducted 5000 permutation tests to obtain null distributions. Specifically, in a given permutation iteration, we calculate network properties after shuffling the group labels within participants. Permutation *P*-value was calculated by *P* = *K*/5000, where *K* is the number of iterations where the observed difference > the real difference.

#### Control analysis

A symptom network with demographic covariates was estimated to explore potential factors influencing the network structure. In addition, a delta network was calculated as the absolute differences in edge weights between the original symptom network and the network after controlling for covariates.

## Results

### Demographic and psychological characteristics of the students

Among the 1501 participants, 193 entries were excluded, leaving 1308 participants included in the analysis. There was no significant difference in the students’ sociodemographics between the school and vacation groups except for age (Table 1). Self-reported measures are presented in Table 2 and descriptive statistics of the 16 items are presented in Supplemental Table 2. Both the PHQ-9 and GAD-7 questionnaire showed good internal consistency, with Cronbach's alpha reaching 0.860 for the PHQ-9 and 0.917 for the GAD-7 questionnaire. There were 992 (71.9%) participants who reported at least mild depression, and 639 (46.3%) reported at least mild anxiety symptoms at school. During summer vacation, 553 (50.3%) and 253 (23.0%) participants reported at least mild depression and anxiety symptoms, respectively. The depression and anxiety levels significantly reduced in the vacation group compared to the school group (PHQ-9: *Z* = 127.8, *P* < 0.001; GAD-7 questionnaire: *Z* = 155.0, *P* < 0.001; Table 2).

**Table 1:** Demographic and characteristics of the school and vacation groups

Participant characteristics	School (*n* = 1380)	Vacation (*n* = 1100)	*t*/*χ*^2^	*P*-value
Age (mean, s.d.)	13.8 (0.88)	14.2 (0.90)	6.500	<0.001^a^
Gender (*n*, %)			0.036	0.85^b^
Male	763 (56.0)	604 (54.9)		
Female	617 (44.0)	496 (45.1)		
Residence (*n*, %)			0.043	0.85^b^
Urban	972 (67.6)	751 (68.3)		
Rural	408 (32.4)	349 (31.7)		
Only child (*n*, %)			0.001	0.98^b^
Yes	446 (32.3)	356 (32.4)		
No	934 (67.7)	744 (67.6)		
Grade (*n*, %)			0.476	0.79^b^
7	412 (29.9)	325 (29.5)		
8	403 (29.2)	335 (30.5)		
9	565 (40.9)	440 (40.0)		
Monthly household income per capita (CNY) (*n*, %)			4.275	0.37^b^
<1000	61 (4.1)	40 (4.2)		
1000–5000	482 (35.8)	350 (35.3)		
5000–10 000	528 (38.2)	449 (38.0)		
10 000–50 000	277 (19.8)	236 (20.2)		
>50 000	32 (2.1)	25 (2.3)		
Educational level of father (*n*, %)			1.116	0.89^b^
Primary school or below	76 (5.5)	60 (5.5)		
Middle school	590 (42.8)	465 (42.3)		
High school	440 (31.9)	340 (30.9)		
Bachelor	236 (17.1)	199 (18.1)		
Master or above	38 (2.7)	36 (3.2)		
Educational level of mother (*n*, %)			1.498	0.83^b^
Primary school or below	90 (6.5)	73 (6.6)		
Middle school	604 (43.8)	474 (43.1)		
High school	417 (30.2)	319 (29.0)		
Bachelor	231 (16.7)	198 (18.0)		
Master or above	38 (2.8)	36 (3.3)		

a. *P-*values obtained by Student's *t*-test.

b. *P-*values obtained by Pearson's *χ*^2^ test.

**Table 2:** Depression and anxiety during school and vacation

Participant characteristics	School (*n* = 1380)	Vacation (*n* = 1100)	*Z*/*χ*^2^	*P*-value
PHQ-9				
Score (median, IQR)	7 (4–10)	5 (1–8)	127.8	<0.001^a^
Normal (*n*, %)	388 (28.1)	547 (49.7)	10.73	<0.001^b^
Mild (*n*, %)	587 (42.5)	333 (30.3)		
Moderate (*n*, %)	312 (22.6)	149 (13.5)		
Severe (*n*, %)	93 (6.7)	71 (6.5)		
GAD-7 questionnaire				
Score (median, IQR)	6 (2–9)	2 (0–6)	155.0	<0.001^a^
Normal (*n*, %)	741 (53.7)	847 (77.0)	14.74	<0.001^b^
Mild (*n*, %)	413 (29.9)	174 (15.8)		
Moderate (*n*, %)	175 (12.7)	45 (4.1)		
Severe (*n*, %)	51 (3.7)	34 (3.1)		

PHQ-9, Patient Health Questionnaire-9; IQR, interquartile range; GAD-7, Generalized Anxiety Disorder 7-item.

a. *P-*values obtained by Mann–Whitney *U*-test.

b. *P-*values obtained by Pearson's *χ*^2^ test.

PHQ-9 and GAD-7 questionnaire scores were reported as raw numbers.

### Results of multilevel mixed-effect models

MLMs detected a significant effect of group (school versus vacation) on depression (*b* = −1.838, *P* < 0.001) and anxiety (*b* = −2.801, *P* < 0.001) levels. In addition, MLM analysis revealed that being female was significantly associated with higher depression and anxiety levels. In particular, adolescents whose mothers had an educational level of primary school or below exhibited significantly higher depression scores compared to those whose mothers had an educational level of middle school or high school. In addition, adolescents whose mothers had a middle school education level showed higher depression scores than those whose mothers had a high school education level (Supplemental Table 3). Other factors did not demonstrate significant effects.

**Fig. 1:**
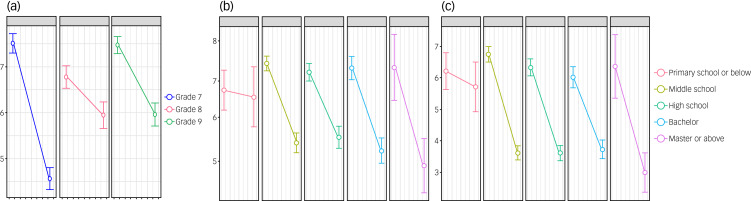
(a) Patient Health Questionnaire-9 (PHQ-9) scores in different grade groups during school and vacation. (b) PHQ-9 in different father education groups during school and vacation. (c). Generalized Anxiety Disorder 7-item questionnaire in different father education groups during school and vacation.

By adding interactive terms, we observed a significant interaction effect between grade and group, and between the father's educational level and group on depression level (Supplemental Table 4). Specifically, the decrease in depression scores in seventh grade was significantly higher than that in other groups (Supplemental Table 4, Fig. 1(a)). In addition, adolescents whose fathers had an educational level of primary school or below exhibited a significantly lower decrease in depression scores during vacation compared to those in other groups (Supplemental Table 4, Fig. 1(b)). Similarly, there was a significant interaction effect between the father's educational level and group on anxiety level, with adolescents whose fathers had an educational level of primary school or below exhibiting a significantly lower decrease in anxiety scores during vacation than those in other groups (Supplemental Table 4, Fig. 1(c)).

### Network estimation and stability analysis

**Fig. 2:**
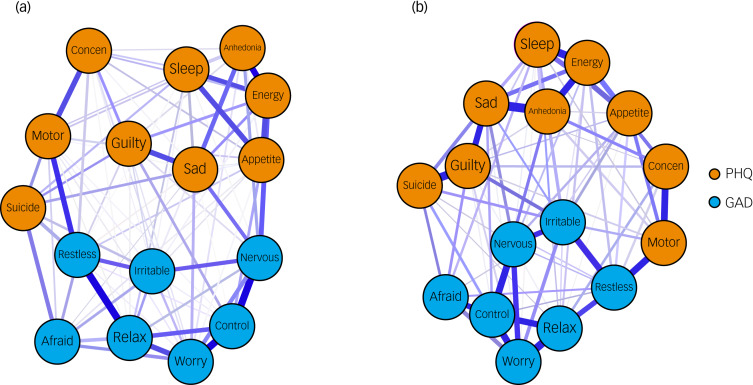
Estimated depression and anxiety symptom network during school (a) and vacation (b). The orange nodes denote the Patient Health Questionnaire-9 (PHQ-9) items and the blue nodes denote the Generalized Anxiety Disorder 7-item (GAD-7) questionnaire items. Concen, concentration.

We constructed depression and anxiety symptom networks at both time points. Symptom network structure plots during school and summer vacations are presented in Fig. 2. Edge weights are listed in Supplemental Table 5 and 6. There were 94 out of 120 (78.3%) possible edges in the school network and 91 out of 120 (75.8%) in the vacation network. Overall, the depression and anxiety symptoms were positively correlated with each other. The symptoms were more densely connected within items from the same scale. Stability analyses revealed that central indices and edge weights were stable in both networks (Supplemental Figs. 1 and 2). Difference tests for centrality indices and edge weights of the networks are displayed in Supplementary Figs. 3 and 4.

### Network centrality indices

Centrality indices are shown in Supplementary Figs. 5 and 7. As for central symptoms, ‘Control Worry’, ‘Restless’, ‘Worry A Lot’, ‘Relax’, ‘Energy’ and ‘Nervous’ exhibited high nodal strength in the school group (nodal strength >1). Meanwhile, symptoms including ‘Control Worry’, ‘Relax’ and ‘Nervous’ consistently showed high nodal strength in the vacation group. As for bridge symptoms, ‘Restless’, ‘Nervous’ and ‘Motor’ had the highest bridge strength in the school group. All three symptoms consistently showed high bridge strength, whereas ‘Irritable’ emerged as the highest bridge strength in the vacation group.

### Network comparison

**Fig. 3:**
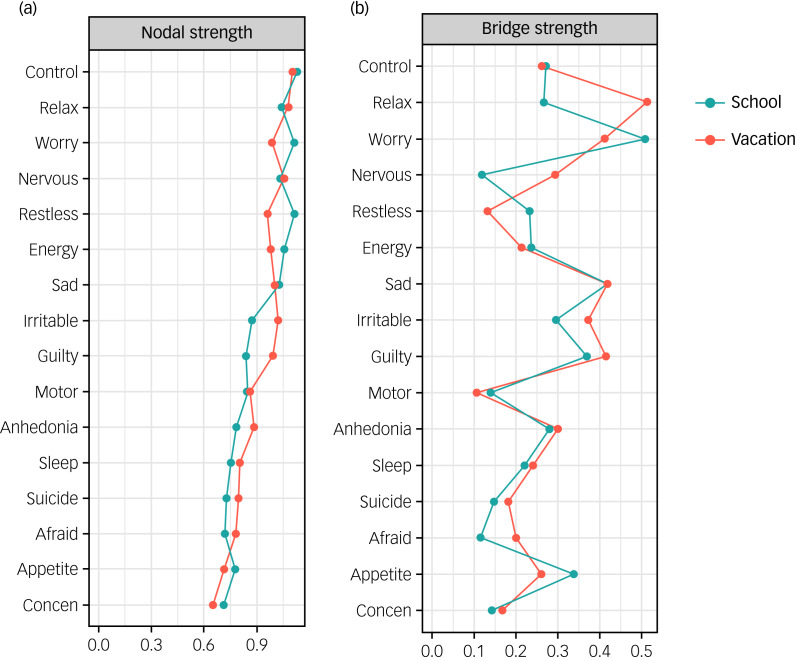
(a) Nodal strength comparison during school and vacation. (b) Bridge strength comparison during school and vacation. Concen, concentration.

Results of the NCT comparing network properties are plotted in Fig. 3 and detailed in Supplemental Table 7. There was no significant difference in the global network strength between school (global network strength 8.10) and vacation (global network strength 8.34) networks (*P* = 0.078). However, the vacation group exhibited higher nodal strength in ‘Guilty’ (*P* = 0.010) and ‘Irritable’ (*P* = 0.010), and lower nodal strength in ‘Worry A Lot’ (*P* = 0.025) and ‘Restless’ (*P* = 0.016) compared to the school group (Fig. 3(a), Supplemental Table 7). The vacation group had significantly higher bridge strength in ‘Relax’ (*P* = 0.020) and ‘Irritable’ (*P* = 0.001) (Fig. 3(b), Supplemental Table 7). No significant difference was detected in the edge weights between school and vacation groups after the false discovery rate (FDR) correction.

### Network with other covariates

To investigate the influence of demographic covariates on our networks, we reconstructed the networks by including seven additional demographic factors. Network plots incorporating covariates are presented in Supplementary Fig. 6. Results demonstrated a significant association between gender and various symptoms, including ‘Sad Mood’ and ‘Nervous’ during school, and ‘Sad Mood’, ‘Energy’ and ‘Worry’ during vacation, with girls having higher levels of the symptoms than boys. Furthermore, age was positively correlated with ‘Anhedonia’ and ‘Nervous’ during school, and ‘Control’ and ‘Sleep’ during vacation. In addition, residence type was correlated with ‘Concentration’ during both school and vacation periods. The delta networks were calculated and are presented in Supplementary Tables 8 and 9. Overall, all the edges in the delta networks are nearly zero, with the strongest edge weights of 0.02 and 0.01 in the baseline delta network and the follow-up delta network, respectively, suggesting that the covariates did not significantly factor into our network models.

## Discussion

In this study, we are principally interested in how students respond to changes in key external environmental factors (i.e. from school to summer vacation). The prevalence and associated factors of depression and anxiety symptoms in adolescent students across school and summer vacation were investigated and differences in the symptom network structures of depression and anxiety between two time points were estimated. Our results suggested that the school–vacation transition had significant impacts on anxiety and depression levels, with certain groups (i.e. students in seventh grade and whose fathers had lower educational levels) being differentially affected. In addition, our study identified that being female and higher age were significant risk factors. Results of network analysis showed that anxiety symptoms were the central symptoms and psychomotor disturbance was the bridge symptom at both times. Moreover, the central and bridge symptoms varied across school and vacation, suggesting that the school–vacation transition also had a significant effect on the depression–anxiety network in adolescents. Altogether, these results indicated that school–vacation transitions could significantly affect depression and anxiety in adolescents. As such, more attention from teachers, schools and families is required for a timely identification of such an impact, as well as for the development of appropriate prevention strategies during school and vacation periods.

### Prevalence and associated factors related to depression and anxiety among adolescents

Our study found that the prevalence of depression and anxiety among adolescent students was very high, with 71.9 and 46.3% of participants exhibiting depression and anxiety symptoms during school, respectively. Moreover, 29.3 and 16.4% of participants met the cut-off score for probable depression and anxiety diagnosis. During the summer vacation, the depression and anxiety levels significantly decreased, with 50.3 and 23.0% having depressive and anxiety symptoms, respectively, and 16.4 and 7.2% having provisional depression and anxiety disorder, respectively. The identified high prevalence of depression and anxiety symptoms during the school year in adolescents may be relevant to the increased academic stress during the school year.^25^ In addition to academic stress, COVID-19 may also affect these two emotions. However, we observed a steady increase in the number of COVID-19 infections from April to August 2021,^26^ and it is observed that depression and anxiety are more likely to worsen as infections escalate.^27^ Consistent with prior research conducted during holiday and epidemic periods, our study reveals that academic pressures significantly affect the mental health of Chinese adolescents, potentially surpassing the influence of the epidemic,^28^ suggesting that the impact from pandemics may be comparatively smaller in contrast to school–vacation transitions. In addition, our study identified several risk factors for depression and anxiety among adolescents in longitudinal assessments. Specifically, adolescent girls are more susceptible to both depression and anxiety than adolescent boys, while higher age was associated with greater severity of depression in this population. Furthermore, we observed that the changes in depression and anxiety symptoms during the vacation vary across different student groups. In particular, students in seventh grade showed a greater reduction in depression and anxiety during vacation than students in other grades, and fathers’ educational level had a significant effect on the symptom change of depression and anxiety. The interactive factors that emerged in our study are in line with some of the previous findings. For example, children with less-educated fathers are more likely to have small academic gains during the summer vacation compared to those with fathers who have higher levels of education, potentially leading to higher levels of stress during vacations.^29^ Altogether, these findings indicate that specific subgroups of adolescents, including girls, older age students and those with parents with lower educational levels, may require increased attention and additional psychological support for depression and anxiety. For instance, the Welsh government in the UK has launched the ‘holiday enrichment programme’ to support children's psychosocial well-being, especially for students from families from low socioeconomic backgrounds.^30^

### Central and bridge symptoms across school–vacation networks

The network structure exhibited different patterns during the school year and summer vacation, suggesting that the school–vacation cycle might have had a marked impact on the presentations of depression and anxiety symptoms. Specifically, we found ‘Control Worry’, ‘Restless’, ‘Worry A Lot’, ‘Trouble Relaxing’ and ‘Nervous’ were the most influential anxiety symptoms in networks during school time, while the results of the NCT revealed that the strength of ‘Restless’ and ‘Worry A Lot’ had decreased during summer vacation, but the other three anxiety symptoms remained the most central symptoms in the vacation network. Consistently, previous studies also identified that anxiety symptoms were the core symptoms in the anxiety–depression network in adolescents.^31^ Meanwhile, we found that ‘Lack of Energy’ was the most central depressive symptom in the network during school time. However, during summer vacation, ‘Guilty’ became the most central depressive symptom in the network.^32^ It might be that schools had posed an excessive burden and sleep restriction on students and further induced the symptom of ‘Lack of energy’,^33^ which then activated additional symptoms in the network, whereas during summer vacation, academic pressure may have been relieved in students. Their psychological stress may come from internalised thoughts such as feeling bad about themselves. Therefore, ‘Guilty’ became more dominant in the network. The above findings illustrate the changing pattern of core symptoms in the depression–anxiety network during school and vacation to some extent.

In terms of bridge symptoms, we found that anxiety symptoms ‘Restless’, ‘Nervous’ and ‘Motor’ were the most important bridge symptoms in the network at both time points. Our results were also in line with previous studies,^34,35^ indicating that the activation of psychomotor symptoms might increase the risk of depression and anxiety. Early intervention targeting these symptoms might help prevent the comorbidity of depression and anxiety among adolescents. During vacation, there was a significantly increased bridge strength in ‘Irritable’ and ‘Trouble Relaxing’, indicating an increased connection between these two symptoms with the depressive symptom cluster. Previous evidence showed that ‘Irritable’ has been correlated with depressive symptoms during the treatment of depression.^36^ Therefore, as one of the anxiety symptoms, ‘Irritable’ could be a prioritised intervention point to relieve depressive symptoms from a symptom comorbidity perspective. In addition, the overall correlation between ‘Trouble Relaxing’ and depressive symptoms was also stronger. Relaxation and recovery during summer vacation have been shown to contribute to the relief of depressive symptoms.^37^ However, further research is necessary to confirm these findings and inform targeted interventions for symptom management.

### Limitations

Several limitations should be acknowledged in our study. First, we did not account for other potentially more specific external stressors during school and summer vacation. As suggested by previous studies, there could be other factors that influence the network structure, such as peer pressure, homework load, parental rearing style, etc.^38,39^ Therefore, to gain a deeper underlying cause of the activation and dynamic changes in the symptom network, it is necessary to detect and account for these potential external factors. Further studies are encouraged to investigate more specific triggering events that produced the activation of the network. A second limitation is that we did not conduct a strict cohort study since all the participants were anonymous in our survey. In addition, we recognise that 20% of the original group of students did not complete the follow-up assessment. Both of these factors could affect the generalisability of the sample. Furthermore, cross-sectional symptom scores may not fully capture the dynamics of psychopathology. The repeated measures of symptoms in each individual over school and vacation periods and a more nuanced statistical approach (e.g. cross-lagged analysis) are warranted in future studies. Third, the simultaneous occurrence of the seasonal transition with the school–vacation cycle may potentially introduce the former as a confounding variable. Future research could explore the impact of the seasonal transition factor by conducting surveys at various holiday time points, such as during the winter holiday period. Besides, our study only included self-reported items from the PHQ-9 and GAD-7 questionnaire, which may introduce response biases; future research should consider incorporating structured clinical assessments and additional data from sources such as parents or schools to obtain a more comprehensive understanding of depression and anxiety symptoms in adolescents. Furthermore, some non-DSM symptoms such as self-hatred and loneliness were the core symptoms of the adolescent depression network, as identified by previous studies.^40^ Future research may also consider including these unique symptoms in a network model.

To sum up, our study revealed a high prevalence of clinically significant depression and anxiety symptoms among adolescent students, especially during the school period. Girls, students of higher age or with lower parental educational levels may require additional psychological support and services. Our findings from the symptom networks indicate that anxiety symptoms might be core symptoms as intervention targets and that psychomotor disturbances may be important targets for preventing depression–anxiety comorbidity. The results also suggested that the depression and anxiety levels as well as the network structure had changed across school and vacation periods, indicating that school–vacation transitions may have an impact on adolescent students’ depression and anxiety symptoms. Taken all together, these findings may inform and provide insights for schools, teachers and family members for supporting children's psychological well-being as well as developing effective early interventions to prevent the emergence of symptoms of depression, anxiety and their comorbidities across school and vacations.

## Supporting information

Ju et al. supplementary material Ju et al. supplementary material

## Data Availability

For more information on the data supporting the analyses and results, please contact the corresponding author, Yan Zhang.
