# Supplementary material for: Examining the effects of school–vacation transitions on depression and anxiety in adolescents: network analysis
Source: BJPsych Open. 2025 Jan 17;11(1):e19. doi: 10.1192/bjo.2024.806 (PMC11795178; doi:10.1192/bjo.2024.806)
Supplement: Ju et al. supplementary material [file S2056472424008068sup001.docx]

**Examining the Effects of School-Vacation Transitions on Depression and Anxiety in Adolescents: A Network Analysis**

***Supplemental Information***

**Network accuracy and stability analysis**

Accuracy of edge weights was evaluated by non-parametric bootstrapping procedure, with smaller bootstrapped CIs indicating higher accuracy of the edge-weights. Stability of the centrality indices was evaluated by case-dropping bootstrapping procedure. Various proportions of cases will be dropped from the dataset at random in each bootstrap procedure, and the correlation was calculated between the original centrality indices and those obtained from subsets. To quantify the stability of centrality indices, correlation stability coefficient (CS-coefficient) was calculated by the maximum proportion of cases that can be dropped to retain a 95% bootstrapped CIs of correlation of ≥ 0.7 between original centrality indices and those from subsamples. Higher CS-coefficient indicates higher stability of the centrality indices. The CS-coefficient ≥ 0.25 indicates acceptable stability and ≥ 0.5 indicates good stability. In addition, the edge weights and centrality indices difference from one another were tested for significance by calculating the difference between bootstrap values of one edge-weight or centrality indices and another edge-weight or centrality indices. A zero in the bootstrapped CI indicates no significant difference between the edge-weights or centrality indices.

Supplementary Figure 1

A. B.


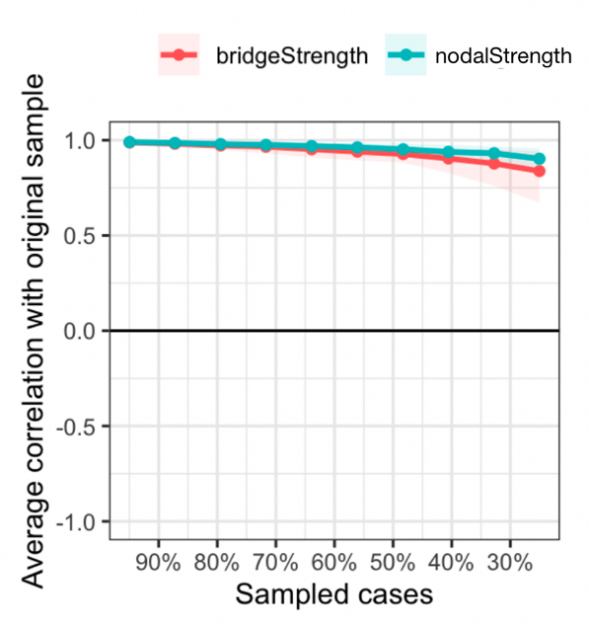

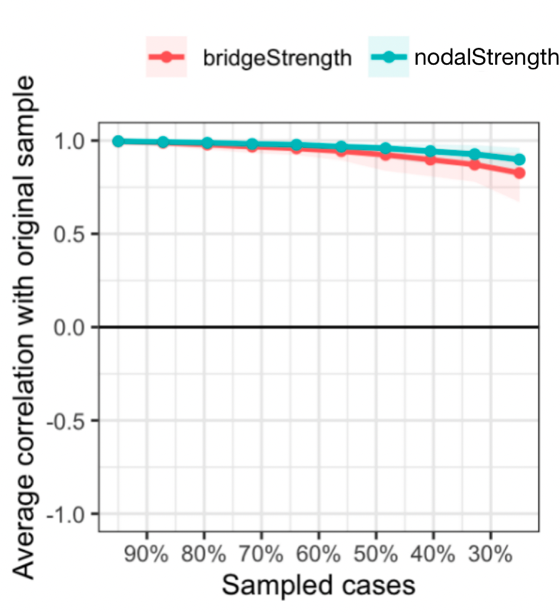


**Fig S1.** Result of stability analyses on strength and bridge strength in school network (A) and vacation network (B). The CS-coefficients for nodal strength and bridge strength in school network were 0.75 and 0.67 respectively. The CS-coefficients for nodal strength and bridge strength in vacation network were 0.75 and 0.67 respectively. Note: The x-axle indicates the proportion of cases included to calculate the centrality indices and the y-axle indicates the correlation between the calculated centrality indices and the original centrality indices. The dots represent the mean value of the calculated centrality indices and the colored plates represent the 95% confidence intervals of the bootstrapped sample.

Supplementary Figure 2

A.


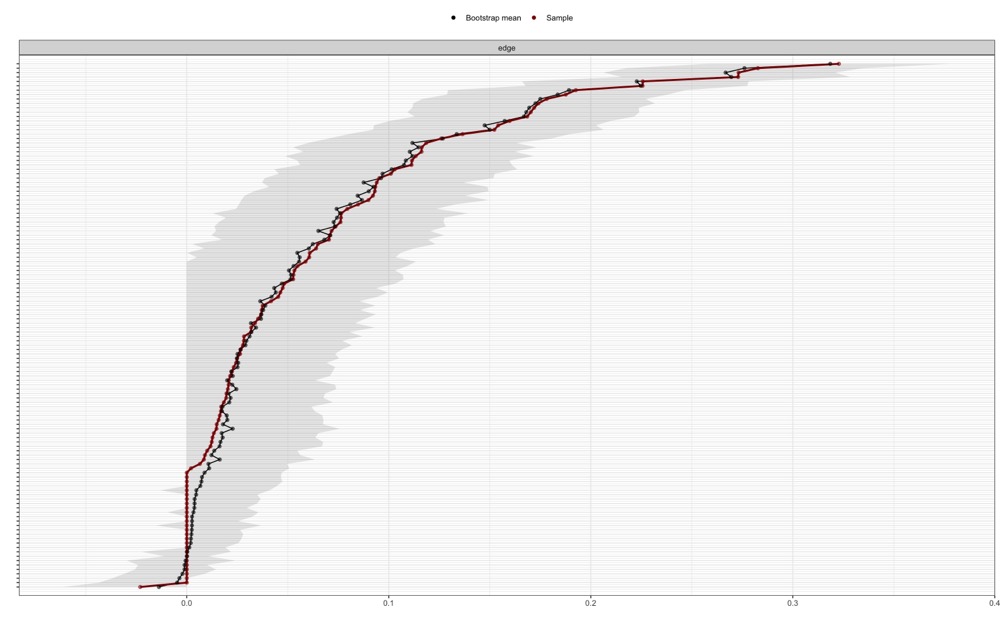


B.


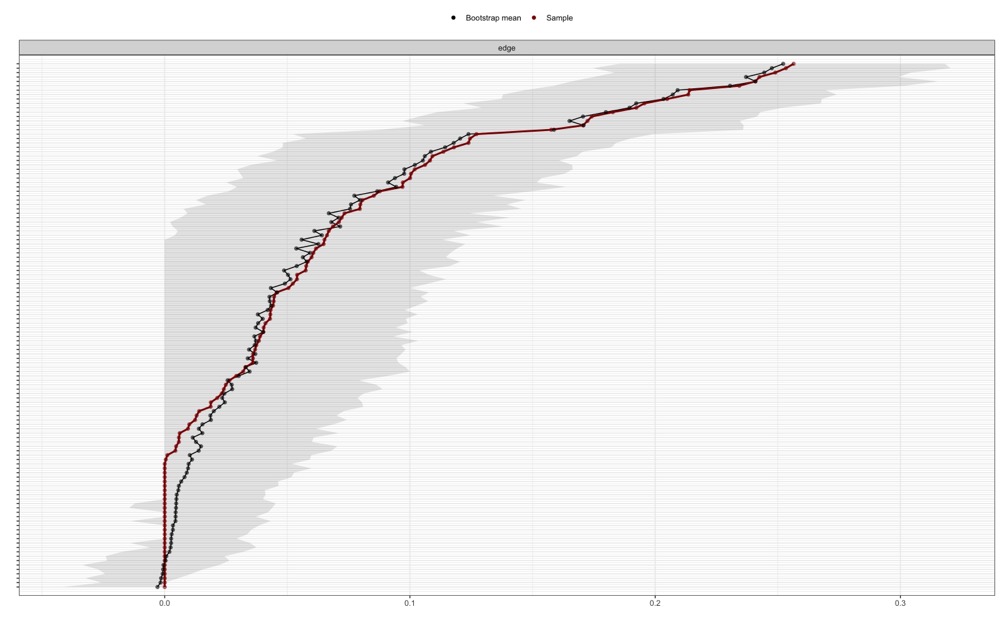


**Fig S2.** Result of edge stability analyses in school network (A) and vacation network (B). Stability analyses revealed that bootstrapping CIs of the edge-weights were stable in both networks. Note: The x-axle indicates the edge weights and the y-axle indicates each edge. The black dots represent the mean value of the bootstrapped edge weights and the red dots represent the edge weights from the original sample. The black lines represent the 95% confidence intervals of the bootstrapped sample.

Supplementary Figure 3

A.


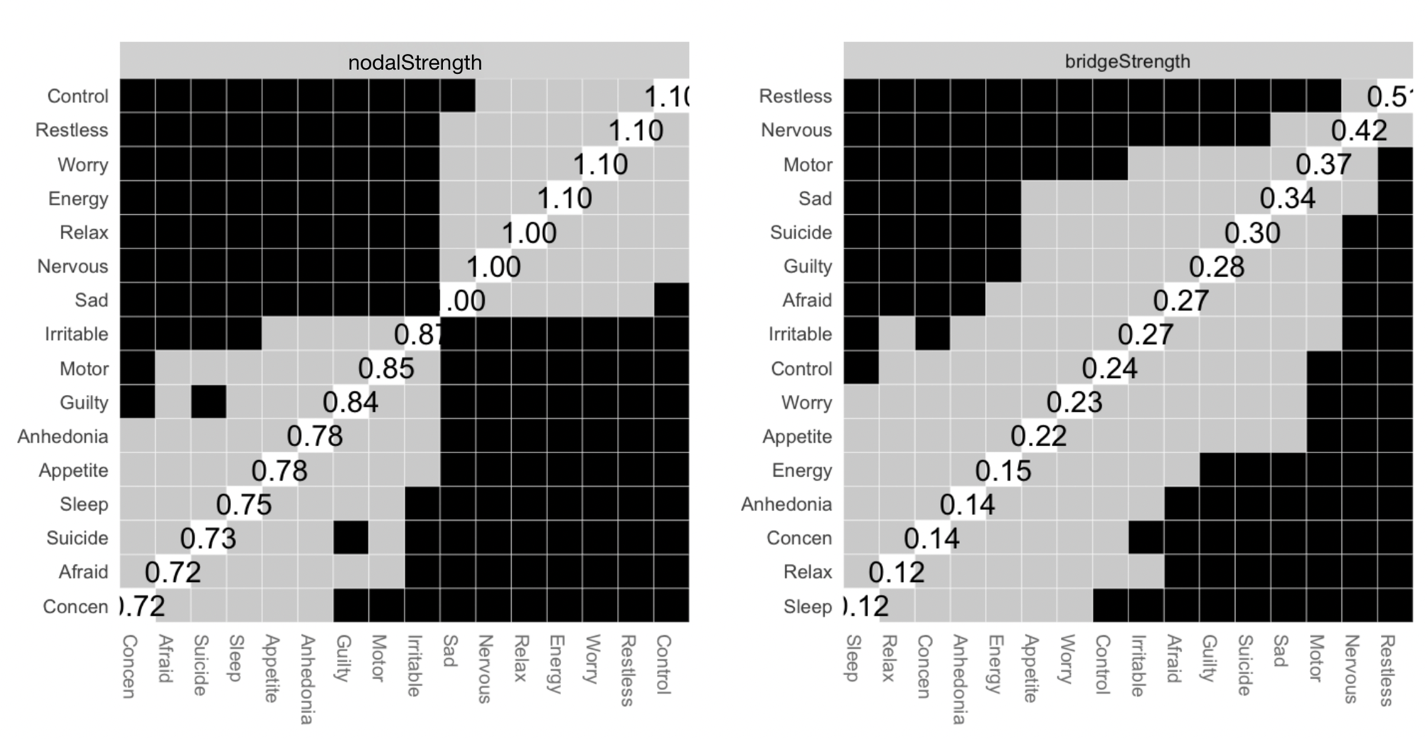
B.


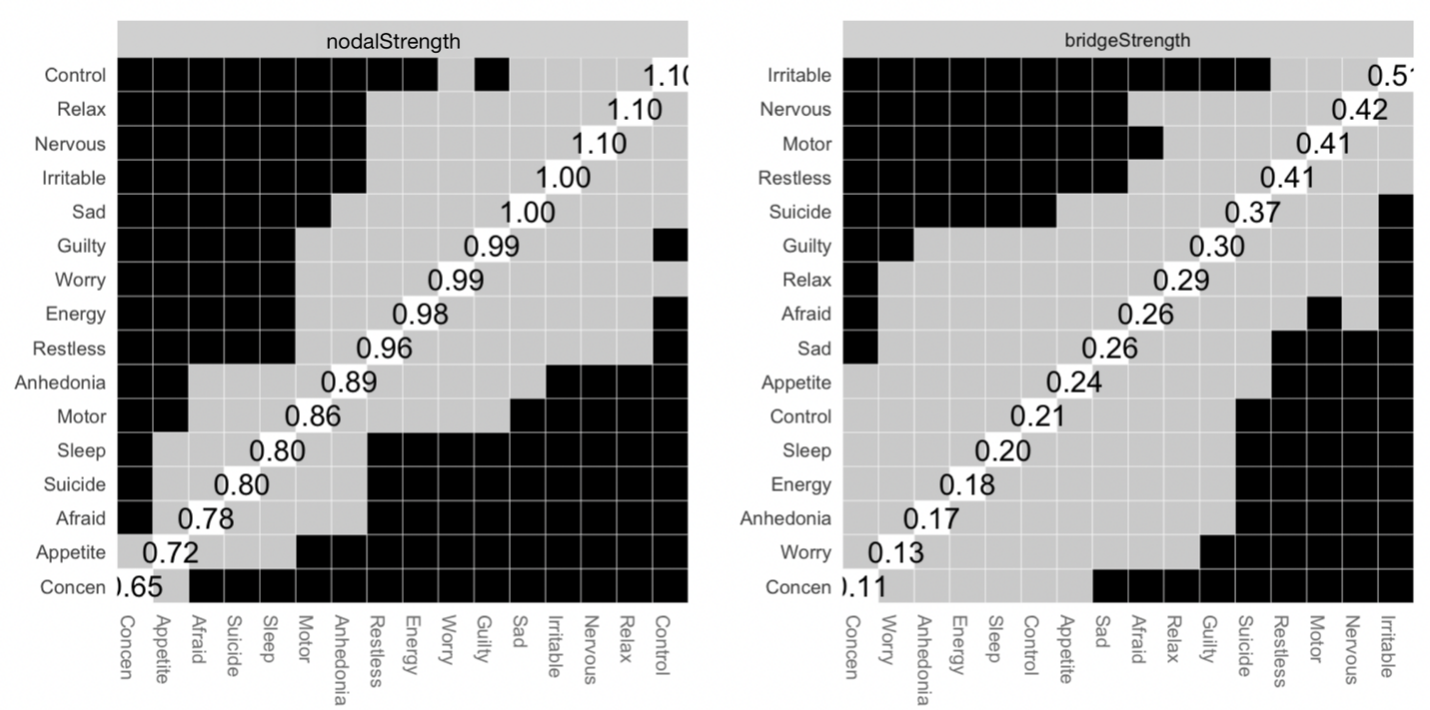
**Fig S3.** Result of difference tests for nodal strength and bridge strength in school network (A) and vacation network (B).

Supplementary Figure 4

A.


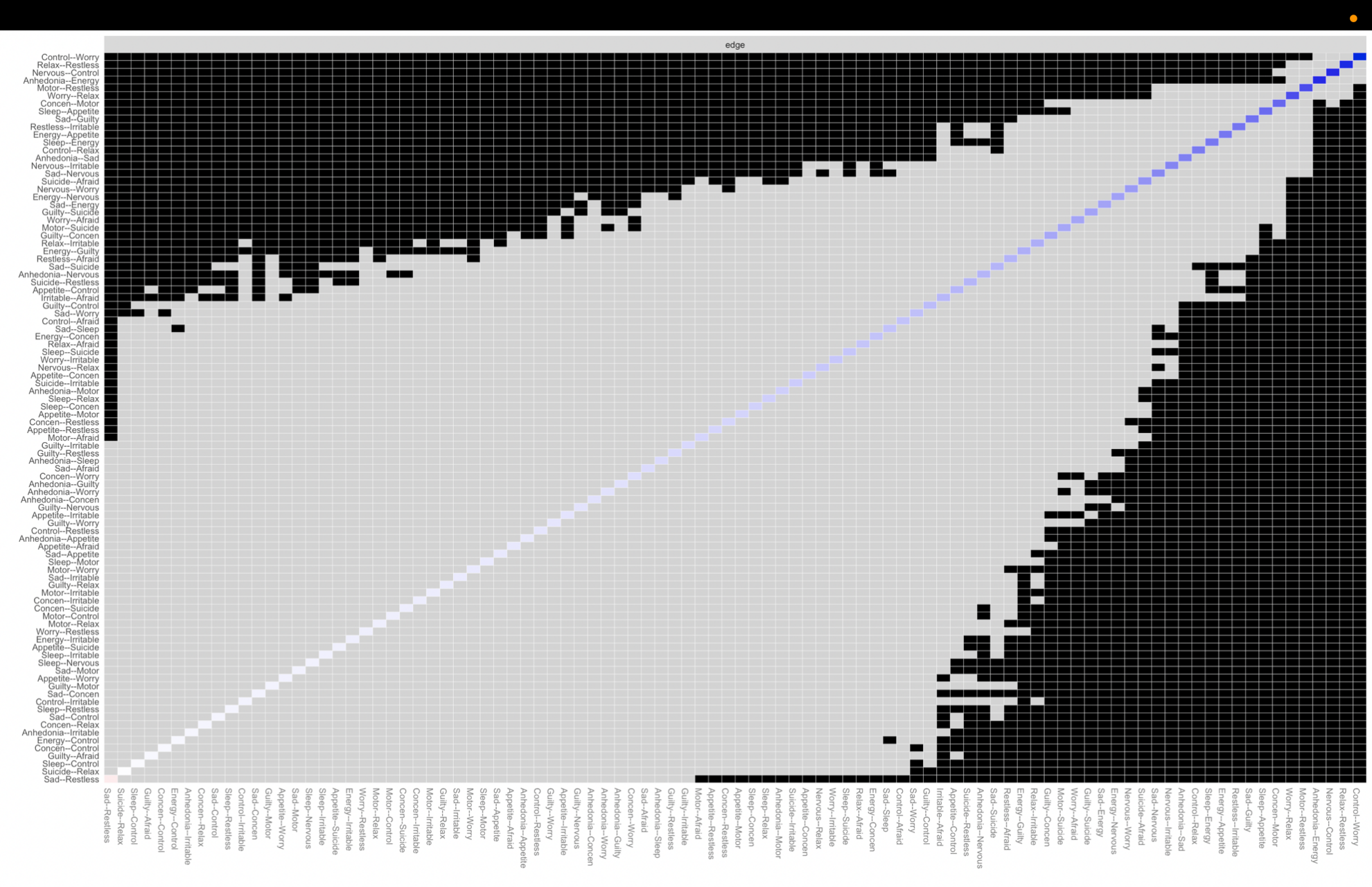


B.
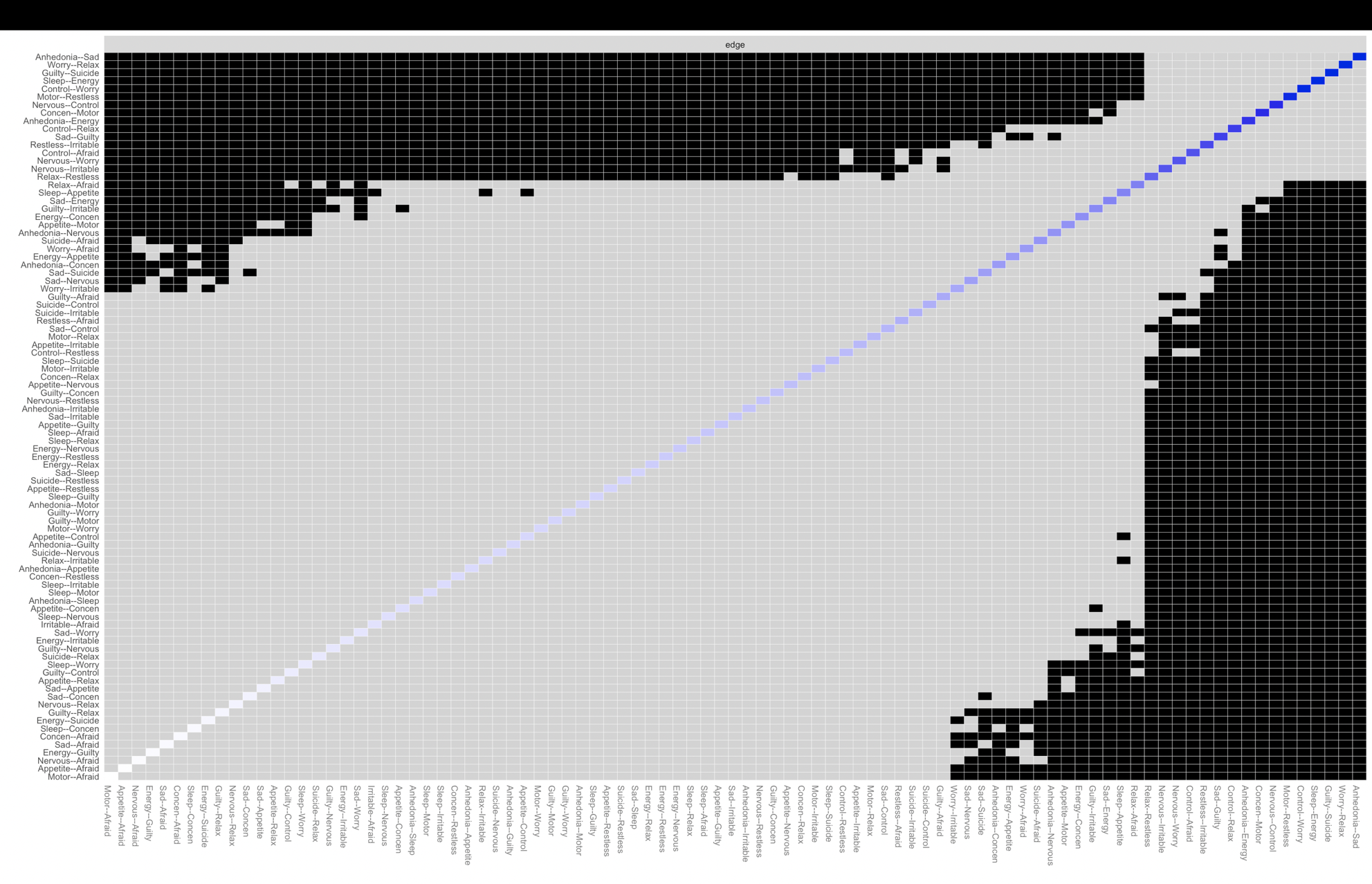


**Fig S4.** Result of difference tests for edge weight in school network (A) and vacation network (B).

Supplementary Figure 5


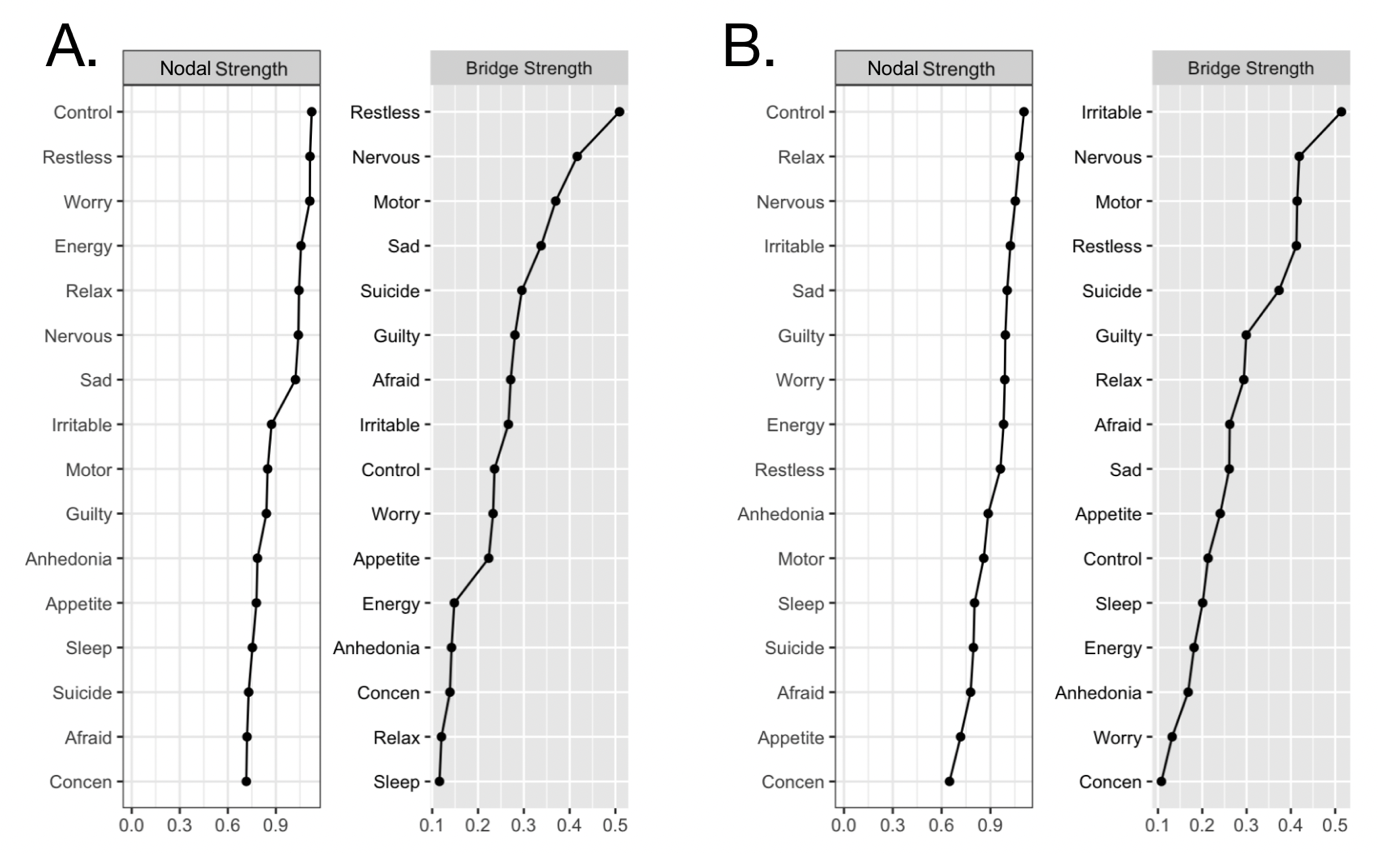


**Fig S5.** Nodal strength and bridge strength estimated for the depression and anxiety symptoms during school (A) and vacation (B).

Supplementary Figure 6


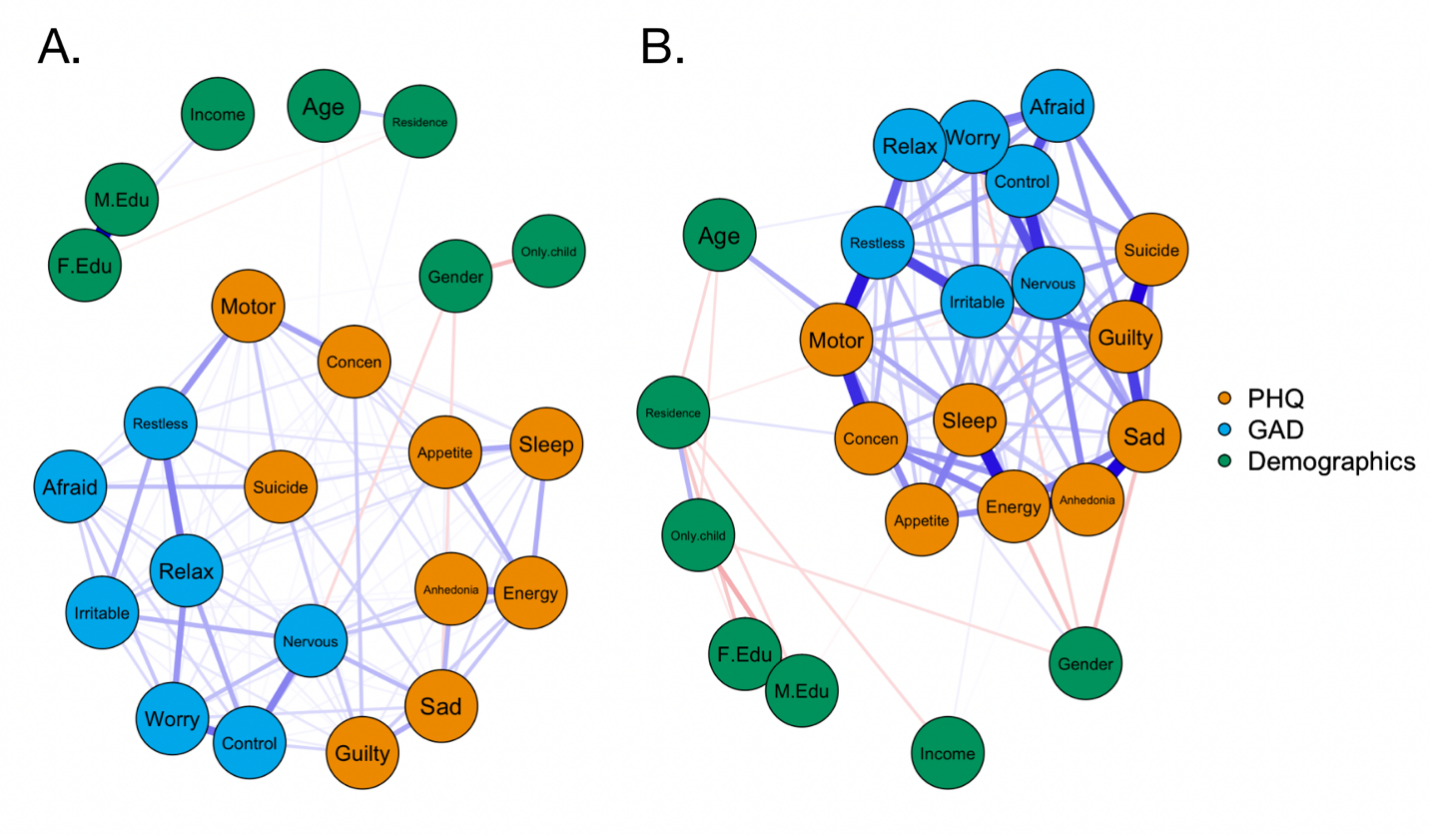


**Fig S6**. Estimated networks after controlling for covariates in school network (A) and vacation network (B).

Table S1. Questionnaires and items used psychological assessments

| Scale | Node Reference Name | Item (Abbreviation) | Item content |
| --- | --- | --- | --- |
| PHQ-9 | Anhedonia | Anhedonia | 1. Little interest or pleasure in doing things |
|  | Sad | Sad Mood | 2. Feeling down, depressed, or hopeless |
|  | Sleep | Sleep | 3. Trouble falling or staying asleep, or sleeping too much |
|  | Energy | Energy | 4. Feeling tired or having little energy |
|  | Appetite | Appetite | 5. Poor appetite or overeating |
|  | Guilty | Guilty | 6. Feeling bad about yourself - or that you are a failure or have let yourself or your family down |
|  | Concen | Concentration | 7. Trouble concentrating on things, such as reading the newspaper or watching television |
|  | Motor | Motor | 8. Moving or speaking so slowly that other people could have noticed. Or so fidgety or restless that you have been moving a lot more than usual |
|  | Suicide | Suicide | 9. Thoughts that you would be better off dead, or thoughts of hurting yourself in some way |
| GAD-7 | Nervous | Nervous | 1. Feeling nervous, anxious, or on edge |
|  | Control | Control Worry | 2. Not being able to stop or control worrying |
|  | Worry | Worry A Lot | 3. Worrying too much about different things |
|  | Relax | Relax | 4. Trouble relaxing |
|  | Restless | Restless | 5. Being so restless that it is hard to sit still |
|  | Irritable | Irritable | 6. Becoming easily annoyed or irritable |
|  | Afraid | Afraid | 7. Feeling afraid, as if something awful might happen |

*Note.* PHQ-9: Patient Health Questionnaire-9; GAD-7: Generalized Anxiety Disorder Scale.

Table S2. Descriptions of the 16 items during school and vacation

| Participant characteristics | School  (n = 1380) | Vacation  (n = 1100) | Z/χ2 | P value |
| --- | --- | --- | --- | --- |
| PHQ-9 (median, IQR) |  |  |  |  |
| Anhedonia | 1 (1-2) | 1 (0-1) | 7.25 | < 0.001 ^a^ |
| Sad Mood | 1 (1-1) | 1 (0-1) | 12.14 | < 0.001 ^a^ |
| Sleep | 1 (0-2) | 0 (0-1) | 5.61 | < 0.001 ^a^ |
| Energy | 1 (1-2) | 1 (0-1) | 11.76 | < 0.001 ^a^ |
| Appetite | 1 (0-2) | 0 (0-1) | 6.67 | < 0.001 ^a^ |
| Guilty | 1 (0-2) | 0 (0-1) | 9.73 | < 0.001 ^a^ |
| Concentration | 1 (0-1) | 0 (0-1) | 6.02 | < 0.001 ^a^ |
| Motor | 0 (0-1) | 0 (0-1) | 4.91 | < 0.001 ^a^ |
| Suicide | 0 (0-1) | 0 (0-0) | 5.95 | < 0.001 ^a^ |
| GAD-7 (median, IQR) |  |  |  |  |
| Nervous | 1 (1-2) | 0 (0-1) | 15.56 | < 0.001 ^a^ |
| Control Worry | 1 (0-2) | 0 (0-1) | 14.80 | < 0.001 ^a^ |
| Worry A Lot | 1 (0-2) | 0 (0-1) | 12.77 | < 0.001 ^a^ |
| Relax | 1 (0-1) | 0 (0-1) | 10.08 | < 0.001 ^a^ |
| Restless | 0 (0-1) | 0 (0-1) | 8.00 | < 0.001 ^a^ |
| Irritable | 1 (0-2) | 0 (0-1) | 7.10 | < 0.001 ^a^ |
| Afraid | 1 (0-1) | 0 (0-1) | 10.72 | < 0.001 ^a^ |

*Note.* PHQ-9: Patient Health Questionnaire-9; GAD-7: Generalized Anxiety Disorder Scale; n, number of participants; a, *P* values obtained by Mann–Whitney U test.

Table S3. Associations of different factors with depressive and anxiety levels (Number of observations =2,480).

| Coefficient | **PHQ-9** | | |  | **GAD-7** | | |
| --- | --- | --- | --- | --- | --- | --- | --- |
|  | *b* | *SE* | *P* |  | *b* | *SE* | *P* |
| Age | 0.412 | 0.232 | 0.076 |  | 0.387 | 0.250 | 0.121 |
| Gender |  |  |  |  |  |  |  |
| Male vs. Female | -1.236 | 0.194 | **< 0.001** |  | -1.258 | 0.209 | **< 0.001** |
| Residence |  |  |  |  |  |  |  |
| Urban vs. Rural | 0.137 | 0.238 | 0.566 |  | -0.520 | 0.486 | 0.285 |
| Only child |  |  |  |  |  |  |  |
| Yes vs. No | -0.199 | 0.210 | 0.343 |  | -0.174 | 0.226 | 0.441 |
| Grade |  |  |  |  |  |  |  |
| 7 vs. 8 | 0.035 | 0.357 | 0.921 |  | 0.155 | 0.372 | 0.678 |
| 8 vs. 9 | -0.146 | 0.509 | 0.774 |  | -0.152 | 0.541 | 0.778 |
| 7 vs. 9 | -0.181 | 0.350 | 0.604 |  | -0.307 | 0.369 | 0.406 |
| Monthly household income per capita |  |  |  |  |  |  |  |
| < 1000 vs. 1000-5000 | 0.007 | 0.526 | 0.990 |  | -0.651 | 0.567 | 0.251 |
| < 1000 vs. 5000-10000 | -0.207 | 0.530 | 0.696 |  | -0.667 | 0.572 | 0.243 |
| < 1000 vs. 10000-50000 | 0.240 | 0.575 | 0.677 |  | -0.130 | 0.62 | 0.834 |
| < 1000 vs. > 50000 | 0.927 | 0.839 | 0.269 |  | 0.644 | 0.905 | 0.477 |
| 1000-5000 vs. 5000-10000 | -0.214 | 0.225 | 0.342 |  | -0.016 | 0.243 | 0.947 |
| 1000-5000 vs. 10000-50000 | 0.233 | 0.288 | 0.418 |  | 0.521 | 0.311 | 0.093 |
| 1000-5000 vs. > 50000 | 0.920 | 0.674 | 0.172 |  | 1.296 | 0.727 | 0.075 |
| 5000-10000 vs. 10000-50000 | 0.447 | 0.271 | 0.098 |  | 0.537 | 0.292 | 0.066 |
| 5000-10000 vs. > 50000 | 1.134 | 0.666 | 0.089 |  | 1.312 | 0.719 | 0.068 |
| 10000-50000 vs. > 50000 | 0.687 | 0.677 | 0.310 |  | 0.774 | 0.73 | 0.289 |
| Educational level of father |  |  |  |  |  |  |  |
| Primary school or below vs. Middle school | 0.121 | 0.450 | 0.788 |  | -0.52 | 0.486 | 0.285 |
| Primary school or below vs. High school | 0.343 | 0.483 | 0.477 |  | -0.68 | 0.521 | 0.192 |
| Primary school or below vs. Bachelor | -0.054 | 0.542 | 0.921 |  | -0.928 | 0.585 | 0.113 |
| Primary school or below vs. Master or above | -0.346 | 0.840 | 0.680 |  | -0.95 | 0.905 | 0.294 |
| Middle school vs. High school | 0.222 | 0.258 | 0.389 |  | -0.160 | 0.279 | 0.565 |
| Middle school vs. Bachelor | -0.175 | 0.351 | 0.618 |  | -0.409 | 0.378 | 0.280 |
| Middle school vs. Master or above | -0.467 | 0.731 | 0.523 |  | -0.430 | 0.788 | 0.585 |
| High school vs. Bachelor | -0.397 | 0.329 | 0.227 |  | -0.307 | 0.369 | 0.406 |
| High school vs. Master or above | -0.689 | 0.718 | 0.337 |  | -0.155 | 0.372 | 0.678 |
| Bachelor vs. Master or above | -0.292 | 0.686 | 0.670 |  | -0.021 | 0.740 | 0.977 |
| Educational level of mother |  |  |  |  |  |  |  |
| Primary school or below vs. Middle school | -0.797 | 0.401 | **0.047** |  | -0.517 | 0.433 | 0.232 |
| Primary school or below vs. High school | -1.313 | 0.439 | **0.003** |  | -0.646 | 0.473 | 0.172 |
| Primary school or below vs. Bachelor | -0.821 | 0.512 | 0.109 |  | -0.662 | 0.552 | 0.230 |
| Primary school or below vs. Master or above | -0.547 | 0.870 | 0.530 |  | -0.819 | 0.939 | 0.383 |
| Middle school vs. High school | -0.517 | 0.260 | **0.047** |  | -0.129 | 0.281 | 0.645 |
| Middle school vs. Bachelor | -0.024 | 0.368 | 0.948 |  | -0.145 | 0.397 | 0.715 |
| Middle school vs. Master or above | 0.250 | 0.796 | 0.754 |  | -0.302 | 0.859 | 0.726 |
| High school vs. Bachelor | 0.493 | 0.343 | 0.152 |  | -0.016 | 0.371 | 0.966 |
| High school vs. Master or above | 0.767 | 0.784 | 0.328 |  | -0.172 | 0.846 | 0.839 |
| Bachelor vs. Master or above | 0.274 | 0.756 | 0.717 |  | -0.156 | 0.816 | 0.848 |
| Group | -2.255 | 0.235 | **< 0.001** |  | -2.996 | 0.241 | **< 0.001** |

Notes: b, unstandardized coefficients; SE, standard error.

Table S4. Associations of different factors with depressive and anxiety levels – MLM further analysis (Number of observations =2,480).

| Coefficient | **PHQ-9** | | |  | **GAD-7** | | |
| --- | --- | --- | --- | --- | --- | --- | --- |
|  | *b* | *SE* | *P* |  | *b* | *SE* | *P* |
| Age | 0.347 | 0.233 | 0.137 |  | 0.367 | 0.250 | 0.142 |
| Gender |  |  |  |  |  |  |  |
| Male vs. Female | -1.191 | 0.194 | **< 0.001** |  | -1.243 | 0.209 | **< 0.001** |
| Residence |  |  |  |  |  |  |  |
| Urban vs. Rural | 0.122 | 0.237 | 0.608 |  | -0.045 | 0.256 | 0.859 |
| Only child |  |  |  |  |  |  |  |
| Yes vs. No | -0.210 | 0.210 | 0.318 |  | -0.185 | 0.227 | 0.414 |
| Grade |  |  |  |  |  |  |  |
| 7 vs. 8 | -2.144 | 0.900 | **0.021** |  | 0.155 | 0.372 | 0.678 |
| 8 vs. 9 | -1.439 | 0.846 | 0.090 |  | -0.152 | 0.541 | 0.778 |
| 7 vs. 9 | 0.705 | 0.854 | 0.412 |  | -0.294 | 0.369 | 0.426 |
| Monthly household income per capita |  |  |  |  |  |  |  |
| < 1000 vs. 1000-5000 | 0.066 | 0.525 | 0.900 |  | -0.584 | 0.568 | 0.303 |
| < 1000 vs. 5000-10000 | -0.109 | 0.530 | 0.838 |  | -0.621 | 0.573 | 0.279 |
| < 1000 vs. 10000-50000 | 0.381 | 0.575 | 0.507 |  | -0.084 | 0.621 | 0.892 |
| < 1000 vs. > 50000 | 1.040 | 0.839 | 0.215 |  | 0.643 | 0.907 | 0.478 |
| 1000-5000 vs. 5000-10000 | -0.175 | 0.226 | 0.440 |  | -0.036 | 0.244 | 0.882 |
| 1000-5000 vs. 10000-50000 | 0.315 | 0.290 | 0.278 |  | 0.500 | 0.313 | 0.110 |
| 1000-5000 vs. > 50000 | 0.974 | 0.675 | 0.149 |  | 1.227 | 0.729 | 0.093 |
| 5000-10000 vs. 10000-50000 | 0.490 | 0.271 | 0.071 |  | 0.537 | 0.293 | 0.067 |
| 5000-10000 vs. > 50000 | 1.149 | 0.666 | 0.085 |  | 1.264 | 0.720 | 0.079 |
| 10000-50000 vs. > 50000 | 0.659 | 0.676 | 0.330 |  | 0.727 | 0.731 | 0.320 |
| Educational level of father |  |  |  |  |  |  |  |
| Primary school or below vs. Middle school | 4.071 | 1.331 | **0.002** |  | 2.690 | 1.439 | 0.062 |
| Primary school or below vs. High school | 3.968 | 1.381 | **0.004** |  | 1.952 | 1.494 | 0.191 |
| Primary school or below vs. Bachelor | 4.215 | 1.463 | **0.004** |  | 1.215 | 1.582 | 0.443 |
| Primary school or below vs. Master or above | 4.819 | 2.129 | **0.024** |  | 2.881 | 2.302 | 0.211 |
| Middle school vs. High school | -0.103 | 0.732 | 0.888 |  | -0.738 | 0.791 | 0.351 |
| Middle school vs. Bachelor | 0.144 | 0.881 | 0.871 |  | -1.475 | 0.952 | 0.122 |
| Middle school vs. Master or above | 0.748 | 1.782 | 0.675 |  | 0.191 | 1.926 | 0.921 |
| High school vs. Bachelor | 0.247 | 0.925 | 0.790 |  | -0.737 | 1.000 | 0.461 |
| High school vs. Master or above | 0.851 | 1.797 | 0.636 |  | 0.929 | 1.942 | 0.632 |
| Bachelor vs. Master or above | 0.604 | 1.834 | 0.742 |  | 1.666 | 1.983 | 0.401 |
| Educational level of mother |  |  |  |  |  |  |  |
| Primary school or below vs. Middle school | -0.719 | 0.400 | 0.072 |  | -0.482 | 0.432 | 0.265 |
| Primary school or below vs. High school | -1.225 | 0.438 | **0.005** |  | -0.611 | 0.473 | 0.197 |
| Primary school or below vs. Bachelor | -0.734 | 0.512 | 0.151 |  | -0.574 | 0.553 | 0.300 |
| Primary school or below vs. Master or above | -0.521 | 0.868 | 0.548 |  | -0.793 | 0.939 | 0.398 |
| Middle school vs. High school | -0.505 | 0.260 | 0.052 |  | -0.129 | 0.281 | 0.646 |
| Middle school vs. Bachelor | -0.015 | 0.370 | 0.967 |  | -0.092 | 0.400 | 0.818 |
| Middle school vs. Master or above | 0.198 | 0.795 | 0.803 |  | -0.312 | 0.859 | 0.717 |
| High school vs. Bachelor | 0.490 | 0.347 | 0.158 |  | 0.037 | 0.375 | 0.921 |
| High school vs. Master or above | 0.703 | 0.782 | 0.369 |  | -0.182 | 0.846 | 0.829 |
| Bachelor vs. Master or above | 0.213 | 0.756 | 0.778 |  | -0.220 | 0.817 | 0.788 |
| Group | -0.271 | 0.878 | 0.758 |  | -1.139 | 0.887 | 0.199 |
| Group * Grade |  |  |  |  |  |  |  |
| Group * 7 vs. 8 | 1.424 | 0.569 | **0.015** |  | n.s. | n.s. | n.s. |
| Group * 7 vs. 9 | 0.929 | 0.508 | **0.049** |  | n.s. | n.s. | n.s. |
| Group * 8 vs. 9 | -0.495 | 0.504 | 0.329 |  | n.s. | n.s. | n.s. |
| Group * Educational level of father |  |  |  |  |  |  |  |
| Group * Primary school or below vs. Middle school | -2.692 | 0.861 | **0.002** |  | -2.203 | 0.931 | **0.018** |
| Group * Primary school or below vs. High school | -2.476 | 0.886 | **0.005** |  | -1.812 | 0.958 | **0.048** |
| Group * Primary school or below vs. Bachelor | -2.879 | 0.928 | **0.002** |  | -1.500 | 1.003 | 0.135 |
| Group * Primary school or below vs. Master or above | -3.492 | 1.340 | **0.009** |  | -2.632 | 1.450 | 0.070 |
| Group * Middle school vs. High school | 0.217 | 0.462 | 0.639 |  | 0.391 | 0.499 | 0.433 |
| Group * Middle school vs. Bachelor | -0.187 | 0.537 | 0.728 |  | 0.704 | 0.581 | 0.226 |
| Group * Middle school vs. Master or above | -0.799 | 1.107 | 0.470 |  | -0.429 | 1.198 | 0.720 |
| Group * High school vs. Bachelor | -0.403 | 0.567 | 0.477 |  | 0.312 | 0.613 | 0.610 |
| Group * High school vs. Master or above | -1.016 | 1.117 | 0.363 |  | -0.820 | 1.208 | 0.497 |
| Group * Bachelor vs. Master or above | -0.612 | 1.146 | 0.593 |  | -1.133 | 1.239 | 0.361 |

Notes: b, unstandardized coefficients; SE, standard error; n.s., not significant.

Table S5. Estimated edge weights in school network

|  | **Anhedonia** | **Sad** | **Sleep** | **Energy** | **Appetite** | **Guilty** | **Concen** | **Motor** | **Suicide** | **Nervous** | **Control** | **Worry** | **Relax** | **Restless** | **Irritable** | **Afraid** |
| --- | --- | --- | --- | --- | --- | --- | --- | --- | --- | --- | --- | --- | --- | --- | --- | --- |
| **Anhedonia** | NA | 0.16 | 0.05 | 0.27 | 0.03 | 0.04 | 0.04 | 0.06 | NA | 0.09 | NA | 0.04 | NA | NA | 0.01 | NA |
| **Sad** | 0.16 | NA | 0.08 | 0.12 | 0.03 | 0.18 | 0.01 | 0.02 | 0.09 | 0.15 | 0.01 | 0.08 | NA | -0.02 | 0.02 | 0.05 |
| **Sleep** | 0.05 | 0.08 | NA | 0.17 | 0.19 | NA | 0.06 | 0.03 | 0.07 | 0.02 | 0.01 | NA | 0.06 | 0.01 | 0.02 | NA |
| **Energy** | 0.27 | 0.12 | 0.17 | NA | 0.17 | 0.10 | 0.08 | NA | NA | 0.12 | 0.01 | NA | NA | NA | 0.02 | NA |
| **Appetite** | 0.03 | 0.03 | 0.19 | 0.17 | NA | NA | 0.06 | 0.05 | 0.02 | NA | 0.09 | 0.02 | NA | 0.05 | 0.03 | 0.03 |
| **Guilty** | 0.04 | 0.18 | NA | 0.10 | NA | NA | 0.11 | 0.02 | 0.12 | 0.04 | 0.08 | 0.03 | 0.02 | 0.05 | 0.05 | 0.01 |
| **Concen** | 0.04 | 0.01 | 0.06 | 0.08 | 0.06 | 0.11 | NA | 0.19 | 0.02 | NA | 0.01 | 0.04 | 0.01 | 0.05 | 0.02 | NA |
| **Motor** | 0.06 | 0.02 | 0.03 | NA | 0.05 | 0.02 | 0.19 | NA | 0.11 | NA | 0.02 | 0.03 | 0.02 | 0.23 | 0.02 | 0.05 |
| **Suicide** | NA | 0.09 | 0.07 | NA | 0.02 | 0.12 | 0.02 | 0.11 | NA | NA | NA | NA | NA | 0.09 | 0.06 | 0.14 |
| **Nervous** | 0.09 | 0.15 | 0.02 | 0.12 | NA | 0.04 | NA | NA | NA | NA | 0.27 | 0.13 | 0.07 | NA | 0.15 | NA |
| **Control** | NA | 0.01 | 0.01 | 0.01 | 0.09 | 0.08 | 0.01 | 0.02 | NA | 0.27 | NA | 0.32 | 0.17 | 0.03 | 0.01 | 0.08 |
| **Worry** | 0.04 | 0.08 | NA | NA | 0.02 | 0.03 | 0.04 | 0.03 | NA | 0.13 | 0.32 | NA | 0.23 | 0.02 | 0.07 | 0.11 |
| **Relax** | NA | NA | 0.06 | NA | NA | 0.02 | 0.01 | 0.02 | NA | 0.07 | 0.17 | 0.23 | NA | 0.28 | 0.10 | 0.07 |
| **Restless** | NA | -0.02 | 0.01 | NA | 0.05 | 0.05 | 0.05 | 0.23 | 0.09 | NA | 0.03 | 0.02 | 0.28 | NA | 0.17 | 0.10 |
| **Irritable** | 0.01 | 0.02 | 0.02 | 0.02 | 0.03 | 0.05 | 0.02 | 0.02 | 0.06 | 0.15 | 0.01 | 0.07 | 0.10 | 0.17 | NA | 0.09 |
| **Afraid** | NA | 0.05 | NA | NA | 0.03 | 0.01 | NA | 0.05 | 0.14 | NA | 0.08 | 0.11 | 0.07 | 0.10 | 0.09 | NA |

Table S6. Estimated edge weights in vacation network

|  | **Anhedonia** | **Sad** | **Sleep** | **Energy** | **Appetite** | **Guilty** | **Concen** | **Motor** | **Suicide** | **Nervous** | **Control** | **Worry** | **Relax** | **Restless** | **Irritable** | **Afraid** |
| --- | --- | --- | --- | --- | --- | --- | --- | --- | --- | --- | --- | --- | --- | --- | --- | --- |
| **Anhedonia** | NA | 0.26 | 0.04 | 0.20 | 0.04 | 0.04 | 0.10 | 0.04 | NA | 0.11 | NA | NA | NA | NA | 0.06 | NA |
| **Sad** | 0.26 | NA | 0.05 | 0.12 | 0.01 | 0.19 | 0.01 | NA | 0.10 | 0.10 | 0.07 | 0.03 | NA | NA | 0.06 | 0.01 |
| **Sleep** | 0.04 | 0.05 | NA | 0.24 | 0.12 | 0.04 | 0.01 | 0.04 | 0.07 | 0.03 | NA | 0.02 | 0.05 | NA | 0.04 | 0.06 |
| **Energy** | 0.20 | 0.12 | 0.24 | NA | 0.10 | NA | 0.11 | NA | 0.01 | 0.05 | NA | NA | 0.05 | 0.05 | 0.02 | NA |
| **Appetite** | 0.04 | 0.01 | 0.12 | 0.10 | NA | 0.06 | 0.03 | 0.11 | NA | 0.06 | 0.04 | NA | 0.02 | 0.04 | 0.07 | NA |
| **Guilty** | 0.04 | 0.19 | 0.04 | NA | 0.06 | NA | 0.06 | 0.04 | 0.25 | 0.02 | 0.02 | 0.04 | 0.01 | NA | 0.12 | 0.09 |
| **Concen** | 0.10 | 0.01 | 0.01 | 0.11 | 0.03 | 0.06 | NA | 0.21 | NA | NA | NA | NA | 0.07 | 0.04 | NA | 0.01 |
| **Motor** | 0.04 | NA | 0.04 | NA | 0.11 | 0.04 | 0.21 | NA | NA | NA | NA | 0.04 | 0.07 | 0.23 | 0.07 | NA |
| **Suicide** | NA | 0.10 | 0.07 | 0.01 | NA | 0.25 | NA | NA | NA | 0.04 | 0.08 | NA | 0.02 | 0.04 | 0.08 | 0.11 |
| **Nervous** | 0.11 | 0.10 | 0.03 | 0.05 | 0.06 | 0.02 | NA | NA | 0.04 | NA | 0.21 | 0.17 | 0.01 | 0.06 | 0.17 | NA |
| **Control** | NA | 0.07 | NA | NA | 0.04 | 0.02 | NA | NA | 0.08 | 0.21 | NA | 0.24 | 0.20 | 0.07 | NA | 0.17 |
| **Worry** | NA | 0.03 | 0.02 | NA | NA | 0.04 | NA | 0.04 | NA | 0.17 | 0.24 | NA | 0.25 | NA | 0.09 | 0.10 |
| **Relax** | NA | NA | 0.05 | 0.05 | 0.02 | 0.01 | 0.07 | 0.07 | 0.02 | 0.01 | 0.20 | 0.25 | NA | 0.16 | 0.04 | 0.13 |
| **Restless** | NA | NA | NA | 0.05 | 0.04 | NA | 0.04 | 0.23 | 0.04 | 0.06 | 0.07 | NA | 0.16 | NA | 0.18 | 0.08 |
| **Irritable** | 0.06 | 0.06 | 0.04 | 0.02 | 0.07 | 0.12 | NA | 0.07 | 0.08 | 0.17 | NA | 0.09 | 0.04 | 0.18 | NA | 0.03 |
| **Afraid** | NA | 0.01 | 0.06 | NA | NA | 0.09 | 0.01 | NA | 0.11 | NA | 0.17 | 0.10 | 0.13 | 0.08 | 0.03 | NA |

Table S7. Results of NCT comparing centrality indices between groups

| Items | Nodal strength at school | Nodal strength at vacation | Bridge strength at school | Bridge strength at vacation | *P_1_* | *P_2_* |
| --- | --- | --- | --- | --- | --- | --- |
| 1. Anhedonia (PHQ-1) | 0.78 | 0.89 | 0.14 | 0.17 | 0.10 | 0.60 |
| 2. Sad Mood (PHQ-2) | 1.02 | 1.00 | 0.34 | 0.26 | 0.76 | 0.20 |
| 3. Sleep (PHQ-3) | 0.75 | 0.80 | 0.12 | 0.20 | 0.35 | 0.12 |
| 4. Energy (PHQ-4) | 1.06 | 0.98 | 0.15 | 0.18 | 0.17 | 0.53 |
| 5. Appetite (PHQ-5) | 0.78 | 0.72 | 0.22 | 0.24 | 0.24 | 0.76 |
| 6. Guilty (PHQ-6) | 0.84 | 0.99 | 0.28 | 0.30 | 0.010^**^ | 0.78 |
| 7. Concentration (PHQ-7) | 0.72 | 0.65 | 0.14 | 0.11 | 0.16 | 0.57 |
| 8. Motor (PHQ-8) | 0.85 | 0.86 | 0.37 | 0.41 | 0.87 | 0.47 |
| 9. Suicide (PHQ-9) | 0.73 | 0.80 | 0.30 | 0.37 | 0.23 | 0.23 |
| 10. Nervous (GAD-1) | 1.04 | 1.05 | 0.42 | 0.42 | 0.82 | 0.97 |
| 11. Control Worry (GAD-2) | 1.12 | 1.11 | 0.24 | 0.21 | 0.76 | 0.71 |
| 12. Worry A Lot (GAD-3) | 1.11 | 0.99 | 0.23 | 0.13 | 0.025^*^ | 0.13 |
| 13. Relax (GAD-4) | 1.04 | 1.08 | 0.12 | 0.29 | 0.54 | 0.020^*^ |
| 14. Restless (GAD-5) | 1.11 | 0.96 | 0.51 | 0.41 | 0.016^*^ | 0.21 |
| 15. Irritable (GAD-6) | 0.87 | 1.02 | 0.27 | 0.51 | 0.010^*^ | 0.001^***^ |
| 16. Afraid (GAD-7) | 0.72 | 0.78 | 0.27 | 0.26 | 0.30 | 0.91 |

*Note.* *P_1_*: Nodal strength at school vs. Nodal strength at vacation; *P_2_*: Bridge strength at school vs. Bridge strength at vacation; n, number of participants; ^*^, *P* < 0.05; ^**^, *P* < 0.01; ^***^, *P* < 0.001.

Table S8. Delta edge weights in school network

|  | **Anhedonia** | **Sad** | **Sleep** | **Energy** | **Appetite** | **Guilty** | **Concen** | **Motor** | **Suicide** | **Nervous** | **Control** | **Worry** | **Relax** | **Restless** | **Irritable** | **Afraid** |
| --- | --- | --- | --- | --- | --- | --- | --- | --- | --- | --- | --- | --- | --- | --- | --- | --- |
| **Anhedonia** | 0.000 | 0.006 | 0.000 | 0.013 | 0.002 | 0.002 | 0.001 | 0.002 | 0.001 | 0.000 | 0.000 | 0.003 | 0.000 | 0.000 | 0.001 | 0.000 |
| **Sad** | 0.006 | 0.000 | 0.003 | 0.001 | 0.001 | 0.010 | 0.000 | 0.003 | 0.005 | 0.012 | 0.005 | 0.003 | 0.000 | 0.023 | 0.003 | 0.002 |
| **Sleep** | 0.000 | 0.003 | 0.000 | 0.006 | 0.009 | 0.000 | 0.003 | 0.000 | 0.004 | 0.004 | 0.003 | 0.000 | 0.001 | 0.000 | 0.000 | 0.000 |
| **Energy** | 0.013 | 0.001 | 0.006 | 0.000 | 0.006 | 0.002 | 0.000 | 0.000 | 0.000 | 0.003 | 0.007 | 0.000 | 0.000 | 0.000 | 0.003 | 0.000 |
| **Appetite** | 0.002 | 0.001 | 0.009 | 0.006 | 0.000 | 0.000 | 0.003 | 0.001 | 0.001 | 0.000 | 0.003 | 0.006 | 0.000 | 0.001 | 0.000 | 0.001 |
| **Guilty** | 0.002 | 0.010 | 0.000 | 0.002 | 0.000 | 0.000 | 0.006 | 0.003 | 0.004 | 0.005 | 0.003 | 0.004 | 0.003 | 0.004 | 0.001 | 0.002 |
| **Concen** | 0.001 | 0.000 | 0.003 | 0.000 | 0.003 | 0.006 | 0.000 | 0.010 | 0.000 | 0.000 | 0.003 | 0.000 | 0.003 | 0.000 | 0.000 | 0.000 |
| **Motor** | 0.002 | 0.003 | 0.000 | 0.000 | 0.001 | 0.003 | 0.010 | 0.000 | 0.004 | 0.000 | 0.003 | 0.002 | 0.005 | 0.012 | 0.003 | 0.001 |
| **Suicide** | 0.001 | 0.005 | 0.004 | 0.000 | 0.001 | 0.004 | 0.000 | 0.004 | 0.000 | 0.000 | 0.000 | 0.000 | 0.006 | 0.004 | 0.000 | 0.007 |
| **Nervous** | 0.000 | 0.012 | 0.004 | 0.003 | 0.000 | 0.005 | 0.000 | 0.000 | 0.000 | 0.000 | 0.015 | 0.003 | 0.006 | 0.000 | 0.006 | 0.000 |
| **Control** | 0.000 | 0.005 | 0.003 | 0.007 | 0.003 | 0.003 | 0.003 | 0.003 | 0.000 | 0.015 | 0.000 | 0.019 | 0.003 | 0.004 | 0.007 | 0.002 |
| **Worry** | 0.003 | 0.003 | 0.000 | 0.000 | 0.006 | 0.004 | 0.000 | 0.002 | 0.000 | 0.003 | 0.019 | 0.000 | 0.009 | 0.006 | 0.001 | 0.001 |
| **Relax** | 0.000 | 0.000 | 0.001 | 0.000 | 0.000 | 0.003 | 0.003 | 0.005 | 0.006 | 0.006 | 0.003 | 0.009 | 0.000 | 0.017 | 0.000 | 0.002 |
| **Restless** | 0.000 | 0.023 | 0.000 | 0.000 | 0.001 | 0.004 | 0.000 | 0.012 | 0.004 | 0.000 | 0.004 | 0.006 | 0.017 | 0.000 | 0.008 | 0.002 |
| **Irritable** | 0.001 | 0.003 | 0.000 | 0.003 | 0.000 | 0.001 | 0.000 | 0.003 | 0.000 | 0.006 | 0.007 | 0.001 | 0.000 | 0.008 | 0.000 | 0.002 |
| **Afraid** | 0.000 | 0.002 | 0.000 | 0.000 | 0.001 | 0.002 | 0.000 | 0.001 | 0.007 | 0.000 | 0.002 | 0.001 | 0.002 | 0.002 | 0.002 | 0.000 |

Table S9. Delta edge weights in vacation network

|  | **Anhedonia** | **Sad** | **Sleep** | **Energy** | **Appetite** | **Guilty** | **Concen** | **Motor** | **Suicide** | **Nervous** | **Control** | **Worry** | **Relax** | **Restless** | **Irritable** | **Afraid** |
| --- | --- | --- | --- | --- | --- | --- | --- | --- | --- | --- | --- | --- | --- | --- | --- | --- |
| **Anhedonia** | 0.000 | 0.012 | 0.003 | 0.008 | 0.000 | 0.004 | 0.004 | 0.002 | 0.000 | 0.000 | 0.000 | 0.000 | 0.000 | 0.000 | 0.002 | 0.000 |
| **Sad** | 0.012 | 0.000 | 0.002 | 0.003 | 0.003 | 0.010 | 0.004 | 0.000 | 0.001 | 0.003 | 0.001 | 0.002 | 0.001 | 0.000 | 0.002 | 0.003 |
| **Sleep** | 0.003 | 0.002 | 0.000 | 0.014 | 0.006 | 0.000 | 0.001 | 0.002 | 0.003 | 0.001 | 0.000 | 0.000 | 0.001 | 0.000 | 0.001 | 0.002 |
| **Energy** | 0.008 | 0.003 | 0.014 | 0.000 | 0.003 | 0.004 | 0.004 | 0.000 | 0.003 | 0.001 | 0.000 | 0.003 | 0.000 | 0.002 | 0.002 | 0.000 |
| **Appetite** | 0.000 | 0.003 | 0.006 | 0.003 | 0.000 | 0.002 | 0.001 | 0.005 | 0.000 | 0.001 | 0.002 | 0.000 | 0.002 | 0.001 | 0.001 | 0.000 |
| **Guilty** | 0.004 | 0.010 | 0.000 | 0.004 | 0.002 | 0.000 | 0.002 | 0.001 | 0.012 | 0.003 | 0.005 | 0.001 | 0.003 | 0.000 | 0.004 | 0.003 |
| **Concen** | 0.004 | 0.004 | 0.001 | 0.004 | 0.001 | 0.002 | 0.000 | 0.011 | 0.000 | 0.000 | 0.000 | 0.000 | 0.001 | 0.003 | 0.000 | 0.000 |
| **Motor** | 0.002 | 0.000 | 0.002 | 0.000 | 0.005 | 0.001 | 0.011 | 0.000 | 0.000 | 0.000 | 0.000 | 0.005 | 0.000 | 0.010 | 0.001 | 0.002 |
| **Suicide** | 0.000 | 0.001 | 0.003 | 0.003 | 0.000 | 0.012 | 0.000 | 0.000 | 0.000 | 0.002 | 0.000 | 0.000 | 0.003 | 0.001 | 0.000 | 0.004 |
| **Nervous** | 0.000 | 0.003 | 0.001 | 0.001 | 0.001 | 0.003 | 0.000 | 0.000 | 0.002 | 0.000 | 0.010 | 0.006 | 0.008 | 0.003 | 0.008 | 0.005 |
| **Control** | 0.000 | 0.001 | 0.000 | 0.000 | 0.002 | 0.005 | 0.000 | 0.000 | 0.000 | 0.010 | 0.000 | 0.008 | 0.006 | 0.005 | 0.000 | 0.007 |
| **Worry** | 0.000 | 0.002 | 0.000 | 0.003 | 0.000 | 0.001 | 0.000 | 0.005 | 0.000 | 0.006 | 0.008 | 0.000 | 0.012 | 0.000 | 0.001 | 0.000 |
| **Relax** | 0.000 | 0.001 | 0.001 | 0.000 | 0.002 | 0.003 | 0.001 | 0.000 | 0.003 | 0.008 | 0.006 | 0.012 | 0.000 | 0.006 | 0.003 | 0.003 |
| **Restless** | 0.000 | 0.000 | 0.000 | 0.002 | 0.001 | 0.000 | 0.003 | 0.010 | 0.001 | 0.003 | 0.005 | 0.000 | 0.006 | 0.000 | 0.007 | 0.001 |
| **Irritable** | 0.002 | 0.002 | 0.001 | 0.002 | 0.001 | 0.004 | 0.000 | 0.001 | 0.000 | 0.008 | 0.000 | 0.001 | 0.003 | 0.007 | 0.000 | 0.002 |
| **Afraid** | 0.000 | 0.003 | 0.002 | 0.000 | 0.000 | 0.003 | 0.000 | 0.002 | 0.004 | 0.005 | 0.007 | 0.000 | 0.003 | 0.001 | 0.002 | 0.000 |
